# Structural Properties and Phase Transition of Na Adsorption on Monolayer MoS_2_

**DOI:** 10.1186/s11671-016-1550-2

**Published:** 2016-07-15

**Authors:** Hai He, Pengfei Lu, Liyuan Wu, Chunfang Zhang, Yuxin Song, Pengfei Guan, Shumin Wang

**Affiliations:** State Key Laboratory of Information Photonics and Optical Communications, Ministry of Education, Beijing University of Posts and Telecommunications, PO Box 72, Beijing, 100876 China; Beijing Computational Science Research Center, Beijing, 100084 China; State Key Laboratory of Functional Materials for Informatics, Shanghai Institute of Microsystem and Information Technology, Chinese Academy of Sciences, Shanghai, 200050 China; Photonics Laboratory, Department of Microtechnology and Nanoscience, Chalmers University of Technology, 41296 Gothenburg, Sweden

**Keywords:** First-principles, MoS_2_, Structural stability, Phase transition

## Abstract

First-principles calculations are performed to investigate the structural stability of Na adsorption on 1H and 1T phases of monolayer MoS_2_. Our results demonstrate that it is likely to make the stability of distorted 1T phase of MoS_2_ over the 1H phase through adsorption of Na atoms. The type of distortion depends on the concentration of adsorbed Na atoms and changes from zigzag-like to diamond-like with the increasing of adsorbed Na atom concentrations. Our calculations show that the phase transition from 1H-MoS_2_ to 1T-MoS_2_ can be obtained by Na adsorption. We also calculate the electrochemical properties of Na adsorption on MoS_2_ monolayer. These results indicate that MoS_2_ is one of potential negative electrodes for Na-ion batteries.

## Background

In recent years, the study of transition-metal dichalcogenides (TMDs) has been a topic of current interest due to their layered structure [1, 2]. TMDs exhibit a broad range of properties, which are advantageous for a wide range of applications as high-performance functional nanomaterials [3]. Among them, molybdenum disulfide (MoS_2_) has attracted considerable attention because of its important role in ultrasensitive photodetectors, flexible electronic device, lithium ion battery, field effect transistors, and sodium-ion batteries [4–7]. These applications show high figure of merit in microelectronics, thermoelectrics, and optoelectronics.

Bulk MoS_2_ crystal is an indirect-gap semiconductor, which is built up of atomic layers stacking by weak van der Waals force. It is possible to exfoliate MoS_2_ monolayer from the bulk, owing to the weak van der Waals interaction between these layers [8, 9]. The typical monolayers of TMDs come in two varieties, called H and T phase with trigonal or octahedral prismatic coordination, respectively [10]. Consequently, MoS_2_ monolayers come in two phases, called 1H-MoS_2_ and 1T-MoS_2_ [11]. The 1H-MoS_2_ phase has the space group of P6/mmc and is semiconducting with a direct band gap [12]. The 1T-MoS_2_ phase is metallic and metastable relative to the 1H-MoS_2_ phase [13]. However, stable 1T-MoS_2_ phase can be realized by doping of MoS_2_ with Re atoms [14] and be stabilized by adsorption of Li atoms [15].

Previous studies demonstrated the phase transition between 1H-MoS_2_ and 1T-MoS_2_ in the early lithiation process [16–20]. The charge transfer induced by the adatoms leads to turn 1T-MoS_2_ phase into a stable MoS_2_ phase. The phase transition is the main issue for application in Li-ion batteries [20, 21] and Na-ion batteries [22, 23]. A large amount of experimental and theoretical works on the application of MoS_2_ in Li-ion batteries has emerged in the past years [16–21]. Kan et al. [16] studied possible pathways of structural phase transition between 1H-MoS_2_ and 1T-MoS_2_ by increasing lithium adsorption concentration constantly. Esfahani et al. [18] calculated the H-T transition by adsorption of Li atoms on both sides of the MoS_2_ monolayer. Mortazavi et al. [22] investigated phase transition between 2H-MoS_2_ and 1T-MoS_2_ upon Na intercalation. Li-ion batteries are prime energy storage systems at present in amounts of devices used in our daily lives such as smartphones and laptops. Na-ion batteries are excellent alternatives to Li-ion batteries because of their lower cost and the greater availability. However, to our knowledge, there are few theoretical calculations on Na adsorption on monolayer MoS_2_.

In this work, we perform a comprehensive first-principles study of the electronic structure, adsorption energies, phase transitions, and electrochemical properties for Na-adsorption compounds. All reasonable structure phases of MoS_2_ monolayer are introduced. Our results suggest that it is easily to turn to be octahedral phases by Na-adsorption for 1T-MoS_2_, such as ZT-MoS_2_ with zigzag Mo-Mo chains and DT-MoS_2_ in rhombus-shape with Mo-Mo chains. Furthermore, Na adsorption on the MoS_2_ surface can lead to a structural phase transformation from 1H-MoS_2_ to an octahedral coordinated MoS_2_. Average operating voltages by Na adsorption are calculated. This will be helpful to understand the basic processes involved in monolayer MoS_2_ applied in Na-ion batteries.

## Methods

Our calculations are carried out by using the Vienna Ab-initio Simulation Package (VASP) package [24], which is based on density functional theory (DFT) and plane-wave pseudopotential method. The electron exchange-correlation energy is described in the Perdew-Burke-Ernzerhof (PBE) form for the generalized gradient approximation (GGA) [25]. The cut-off energy is set to be 600 eV for the plane-wave expansion of the wave functions. The Brillouin zone integration is represented by the Monkhorst-Pack k-point scheme with 9 × 9 × 1 and 5 × 5 × 1 grid meshes for the (1 × 1) unit cell and (4 × 4) supercell, respectively. The criterion of convergence of energy is chosen as 10^−5^ eV between two ionic steps, and the maximum force allowed on each atom is 0.01 eV/Å. The vacuum space along the z direction is taken to be more than 15 Å for the both 1H-MoS_2_ and 1T-MoS_2_.

**Fig. 1:**
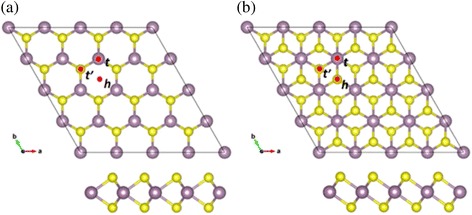
**a** Top and side views of 1H-MoS_2_. **b** Top and side views of 1T-MoS_2_

The geometry structures are shown in Fig. 1. A (4 × 4) supercell of MoS_2_ monolayer consisting of 48 atoms, which contains 16 Mo and 32 S, is made up of the primitive cell of MoS_2_. 1H-MoS_2_ has single S-Mo-S layer, where the Mo site in a trigonal prism coordination as shown in Fig. 1a. 1T-MoS_2_ has asymmetric sulphur atoms sites, where the Mo site in octahedral coordination as shown in Fig. 1b.

## Results and Discussion

### Structural Properties

To obtain a clear insight into the 1H to 1T phase transition, we first calculate electronic structures of both the trigonal prismatic phase (1H-MoS_2_) and octahedral prismatic phase (1T-MoS_2_) by using (1 × 1) unit cell. Our results show that the optimized lattice parameters *a*_0_ = 3.166 Å for both the pristine 1H-MoS_2_ and the pristine 1T-MoS_2_ as shown in Table 1.

**Table 1:** Structural parameters of 1H-MoS_2_ and 1T-MoS_2_ and band gap

		1H-MoS_2_	1T-MoS_2_
a (Å)	Present work	3.166	3.168
	References	3.16 [30]; 3.18 [11]	3.18 [10]
*d*_S-S_ (Å)	Present work	3.092	3.092
	References	3.089 [30]	–
Gap (eV)	Present work	1.71	Metal
	References	1.71 [30]; 1.67 [11]	Metal

**Fig. 2:**
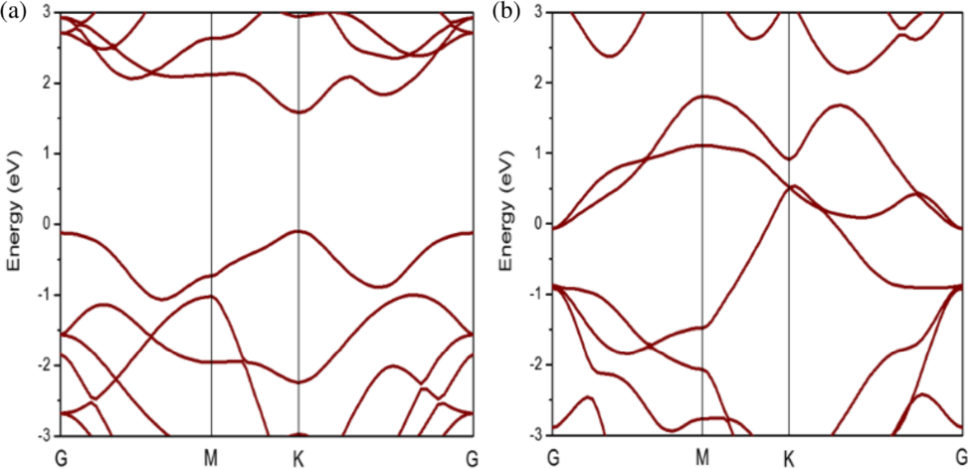
**a** Band structures of 1H-MoS_2_. **b** Band structures of 1T-MoS_2_

Electronic structure provides a clear insight into the difference of band structure between 1H-MoS_2_ and 1T-MoS_2_. The two phases show completely different electronic structures. Figure 2 shows the band structures of 1H-MoS_2_ and 1T-MoS_2_ without spin-orbit coupling. 1H-MoS_2_ is a direct semiconductor with both conduction band minimum (CBM) and valence band maximum (VBM) located at the K point. The band gap obtained from GGA-PBE calculations is 1.71 eV. However, the electronic structure calculation of the 1T structure shows that this polytype is indeed metallic in Fig. 2b. We also calculate the energy difference between 1H-MoS_2_ and undistorted 1T-MoS_2_ unit cell, which shows that the optimized 1H-MoS_2_ is more stable than the 1T-MoS_2_ by 0.84 eV. In normal conditions, although both polytypes of monolayer MoS_2_ have the same element constitution, 1H-MoS_2_ is more stable than 1T-MoS_2_. Besides, the equilibrium lattice constant of 1H-MoS_2_ is close to that of 1T-MoS_2_ according to Table 1.

### Adsorption Energies and Stability Analysis

In order to investigate the stability of the two structural phases with Na adsorption, the most stable configuration of an isolated Na atom adsorbed on (4 × 4) cell for the both structure phases is determined at first. Three different types of adsorption sites are introduced to determine the most stable position [26], including “t” site (top site directly above a Mo atom), t’ site (top site directly above an S atom), and “h” site (hollow site above the center of hexagons), respectively. Na atoms adsorbed at other positions can eventually relax into one of the three listed adsorption sites [27]. Considering the monolayer hexagonal lattice structure of MoS_2_ monolayer, it is reasonable to expect the relaxation of foreign atoms on one of these adsorption sites. There is little change for the adsorption geometry of 1H-MoS_2_ after relaxation. However, the optimized 1T-MoS_2_ supercell will transform into the distorted 1T phase duo to its instability, such as ZT-MoS_2_ with zigzag Mo-Mo chains. Further, to investigate the relative stabilities of the systems, we defined the adsorption energy as follows:

1 $$ {E}_a=\left({E_X}_{-\mathrm{M}\mathrm{o}\mathrm{S}2}+{E}_{\mathrm{Na}}\right)-{E}_{\mathrm{total}}^x $$

where *X* = 1H, distorted 1T, ${E}_{X-\mathrm{M}\mathrm{o}\mathrm{S}2}$ represents the total energy of 1H-MoS_2_ and distorted 1T-MoS_2_ system, ${E}_{\mathrm{total}}^x$ represents the total energy of the adsorption system, and ${E}_{\mathrm{Na}}$ represents the total energy of bulk sodium. The electron configurations of adatom adsorption energies (${E}_a$) and structural properties for single adatom-adsorbed MoS_2_ obtained from our calculations are listed in Table 2. Our calculated results show that the adsorption energy is different for different sites. In all adsorption sites, the site with the largest adsorption energy (minimum total energy) is referred to as the favored one. Comparing the possible sites of h, t, and t’, we found that Na atom prefer to reside on t site for the both structures.

**Table 2:** Adsorption energy, distance between Na and S atoms, the bond length of Mo-Mo

	Site	*E*_a_ (eV)	*d*_Na-S_ (Å)	*d*_Mo-Mo_ (Å)
1H-MoS_2_	h	2.1	2.74	2.97
	t’	3.2	2.68	3.04
	t	1.8	2.76	3.02
Distorted 1T-MoS_2_	h	2.7	2.75	2.89
	t’	3.6	2.66	2.92
	t	2.3	2.76	2.93

### Phase Transition of 2D MoS_2_ Monolayer Induced by Na Insertion

In the previous analysis, we have determined the most stable adsorption site for Na atoms on the surface of MoS_2_ monolayer, which is top of Mo atom sites. Totally, there are 32 most stable sites for Na atoms on both sides of (4 × 4) MoS_2_ supercell [28]. In order to investigate systematically Na adsorption on the surface of MoS_2_ monolayer, we introduce Na atoms on both sides of MoS_2_ monolayer forming the compound 1H-Na_*x*_MoS_2_ and 1T-Na_*x*_MoS_2_ to induce phase transition, which is a solvent-based exfoliation of MoS_2_ monolayer and a typical procedure for both the charge/discharge processes in battery.

**Fig. 3:**
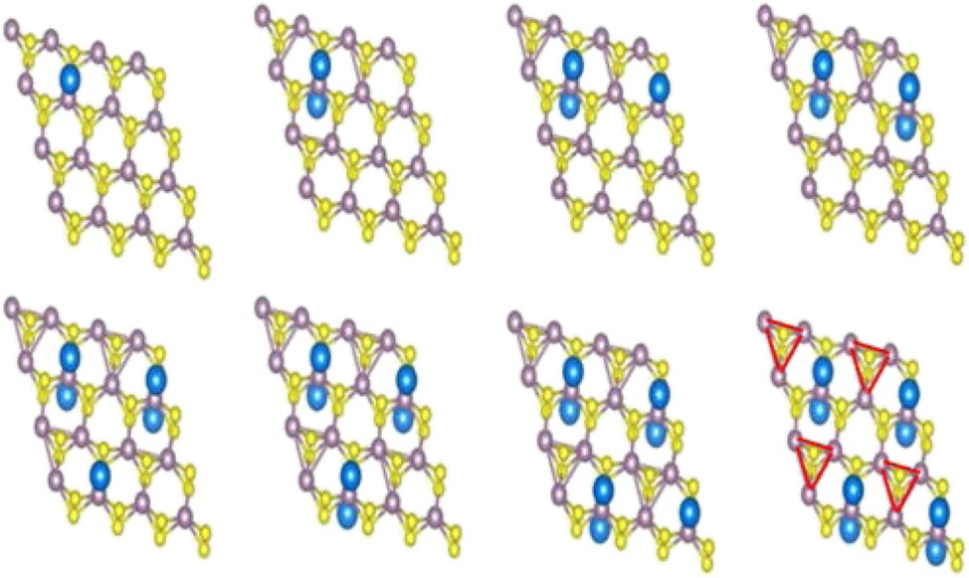
The optimized structures of the most stable 1H-Na_*x*_MoS_2_, triangular Mo-Mo clustering as highlighted in *red*

The geometries of 1H-Na_*x*_MoS_2_ are optimized with adsorption concentration increasing, as shown in Fig. 3. Our results show the variation of energies and structure for 1H-Na_*x*_MoS_2_ with the increasing of Na concentrations. As shown in Fig. 3, Mo-Mo chains appear in 1H-Na_*x*_MoS_2_ when 2~6 Na atoms are added to the system. Triangular Mo-Mo clustering appears in 1H-Na_*x*_MoS_2_ with the increasing of adsorption concentrations.

**Fig. 4:**
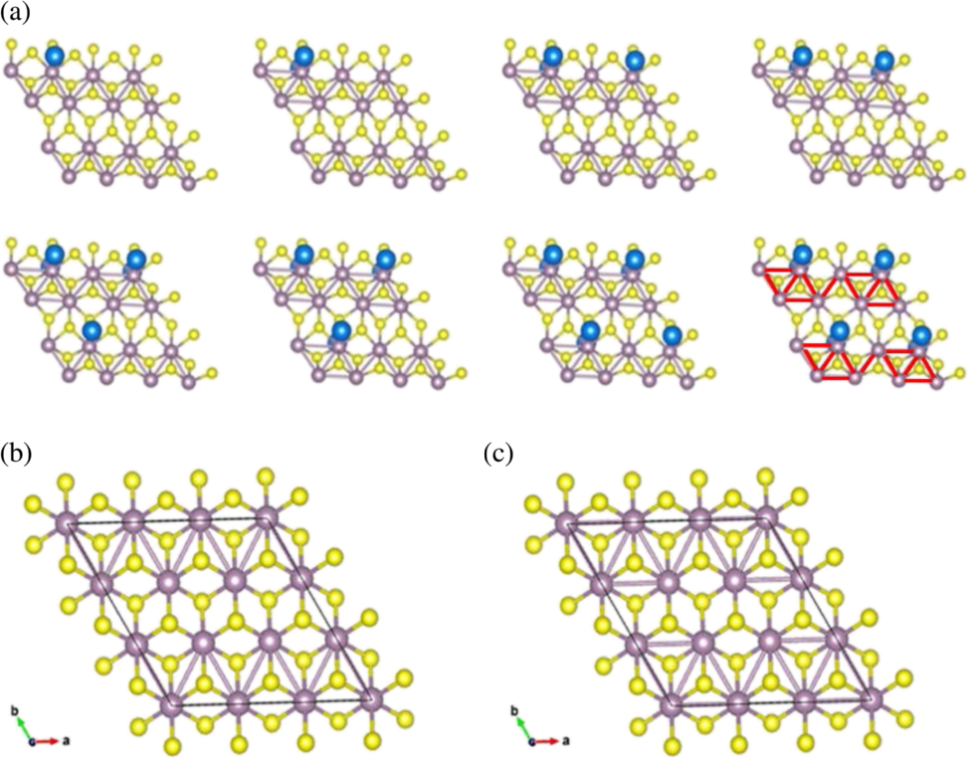
**a** The optimized structures of the most stable 1T-Na_*x*_MoS_2_, diamond-like Mo-Mo clustering as highlighted in *red*. Distorted octahedral coordinated MoS_2_: **b** ZT-MoS_2_: zigzag-like Mo-Mo chains, **c** DT-MoS_2_ diamond-like Mo-Mo chains

The optimized geometries of 1T-Na_*x*_MoS_2_ with the increasing of adsorption concentrations are shown in Fig. 4a. The substrate structure of 1T-MoS_2_ directly transits to the ZT-MoS_2_ after relaxation due to the instability. Some Mo-Mo chains appear in 1T-Na_*x*_MoS_2_ following certain rules. These Mo atoms gradually form a diamond-like chain up to eight Na atoms that are introduced in the system. The system is likely to maintain the diamond chain structure. The geometry configurations of ZT-MoS_2_ and DT-MoS_2_ without adsorption are clearly shown in Fig. 4b, c, respectively. The free-standing 1T-MoS_2_ exhibits metallic property and is metastable. Both ZT-MoS_2_ and DT-MoS_2_ belong to distorted octahedral coordinated MoS_2._

**Fig. 5:**
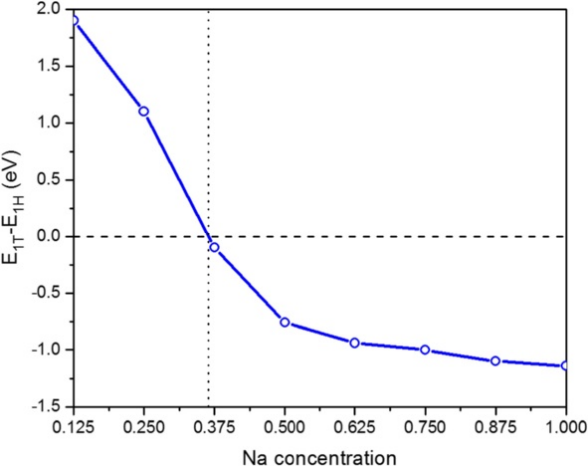
Relative formation energy per Na atom of 1T-Na_*x*_MoS_2_ with respect to 1H-Na_*x*_MoS_2_ as a function of Na concentration

It is similar to the definition of adsorption energy that the formation energy in different concentrations of Na absorption is calculated using the expression:

2 $$ {E}_{f(x)}={E}_{\left(X-\mathrm{N}\mathrm{a}x\mathrm{M}\mathrm{o}\mathrm{S}2\right)}-{E}_{\left(X-\mathrm{M}\mathrm{o}\mathrm{S}2\right)}-n{E}_{\left(\mathrm{N}\mathrm{a}\right)} $$

where *X* = 1H, 1T, ${E}_{\left(X-\mathrm{N}\mathrm{a}x\mathrm{M}\mathrm{o}\mathrm{S}2\right)}$ is the total energy of the X-Na_*x*_MoS_2_ compound, ${E}_{\left(\mathrm{M}\mathrm{o}\mathrm{S}2\right)}$ is the total energy of the same MoS_2_ polytype, and ${E}_{\left(\mathrm{N}\mathrm{a}\right)}$ is the total energy of bulk sodium. A negative binding energy indicates an exothermic chemical interaction between Na and MoS_2_. Relative formation energy per Na atom of 1T-Na_*x*_MoS_2_ with respect to 1H-Na_*x*_MoS_2_ varies as increasing the Na-adsorption concentration constantly in Fig. 5. The 1H-Na_*x*_MoS_2_ still keeps stability in the low adsorption concentration. However, the 1T-Na_*x*_MoS_2_ becomes more stable than 1H-Na_*x*_MoS_2_ when the adsorption concentration of Na atoms exceeds about 35 %. As the adsorption concentration of Na increases, the 1T-Na_*x*_MoS_2_ will become stable further.

### Transition Barrier from 1H Phase to 1T Phase

**Fig. 6:**
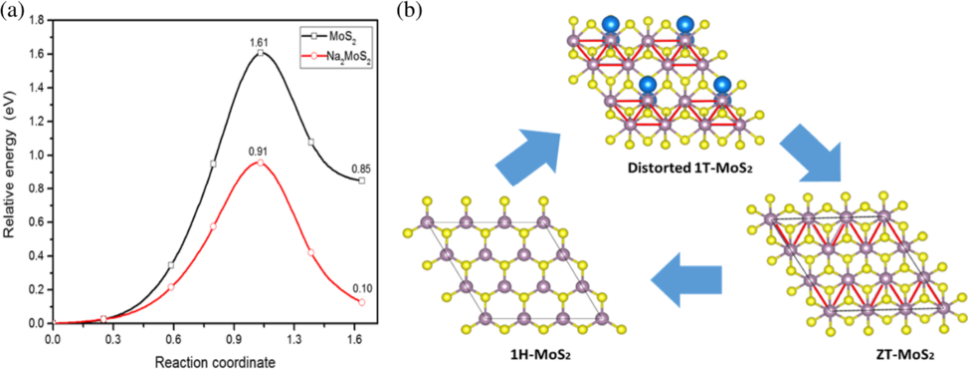
**a** Evolution of the energy per S atom for 1H to 1T structure transition as a function of the reaction coordinate, for pure and Na-covered MoS_2_. **b** The pathways of structural phase transition of Na adsorption on monolayer MoS_2_

The procedure of transition can be viewed as a shift of one S atom layer in the 1H-MoS_2_ structure to the position h in Fig. 1a. Therefore, the barrier energy of 1H-MoS_2_ structure to 1T-MoS_2_ structure transition is able to be calculated by shearing one S layer from the 1H structure toward the 1T structure when fixing the Mo atoms, while the other atoms are allowed to relax. Nudged Elastic Band (NEB) method is adopted to calculate the barrier energy from 1H to 1T structure transition as shown in Fig. 6a. Our results show that the barrier of phase transition from 1H to 1T structure is approximately 1.61 eV in the absence of external adatoms. The phase transition involves one of the S atoms moving from one pyramidal position to the other pyramidal position. The relative energy of Na-intercalated 1T-MoS_2_ is 0.52 eV. Meanwhile, the barrier from 1H-MoS_2_ to 1T-MoS_2_ reduces to 0.91 eV when Na atoms are adsorbed completely on one side of MoS_2_ monolayer. The barrier energy reduces considerably from 1H to 1T structure transition by the Na atom adsorption on MoS_2_ monolayer. These results suggest that the Na atoms are not only effective to make the 1T-MoS_2_ energetically favorable but also play an important role in the process of phase transition. According to our theoretical calculation and experimental works, we summarize the pathways for structural phase transition among different structures in Fig. 6b. The detailed process is the following: (1) When Na atoms are adsorbed to 1H-MoS_2_, the 1H-MoS_2_ remains stable until the Na concentration reaches 35 %. When more Na atoms are adsorbed on both sides of MoS_2_ monolayer, the distorted 1T-MoS_2_ will become more stable. Besides, the structure finally transform to the distorted 1T-MoS_2_ phase with diamond-like chains. (2) When all of the Na atoms are extracted from the system, the structure will become ZT-MoS_2_. (3) The ZT-MoS_2_ will transform back to 1H-MoS_2_ phase by heating or aging.

### Electrochemical Properties of Na_*x*_MoS_2_

**Fig. 7:**
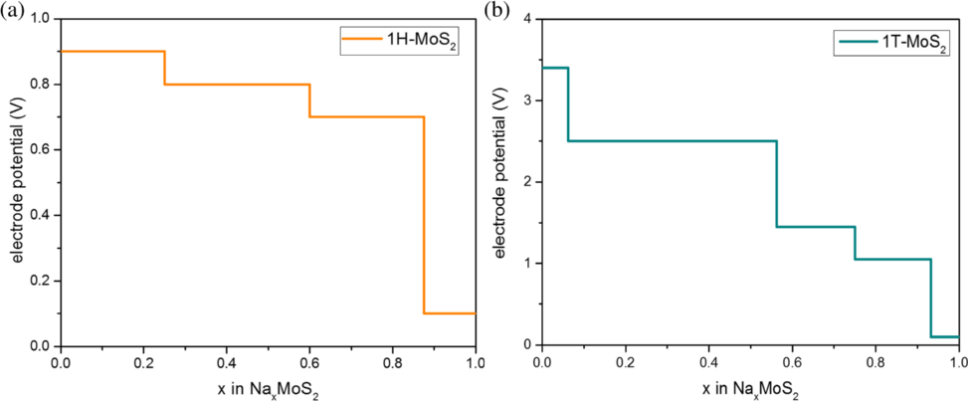
Electrode potential of Na-intercalated **a** 1H-MoS_2_ and **b** 1T-MoS_2_

In order to inspect the suitability of Na_*x*_MoS_2_ compound as an electrode material for Na-ions, we calculate the average adsorption voltage. The electrode potential between Na_*x*1_MoS_2_ and Na_*x*2_MoS_*2*_ (*x*_2_ > *x*_1_) is calculated as [29]:

3 $$ \overline{V}=-\frac{G_{x_2}-{G}_{x_1}-\left({x}_2-{x}_1\right){G}_{Na}}{\left({x}_2-{x}_1\right)e} $$

where ${G}_{x_2}$ and ${G}_{x_1}$ are the total energies of Na_*x*_MoS_2_ systems and ${G}_{\mathrm{Na}}$ is the energy per atom of Na in its bulk state. The electrode potential for 1H-Na_*x*_MoS_2_ and 1T-Na_*x*_MoS_2_ as the change of concentration is shown in Fig. 7, respectively. Our results show that the voltage profile for 1H-Na_*x*_MoS_2_ varies with a decreasing trend in a range of 0~1 V as shown in Fig. 7a, with an average value of 0.72 V. The electrode potential for 1T-Na_*x*_MoS_2_ varies in a range of 0~3.5 V as shown in Fig. 7b. However, 1T-Na_*x*_MoS_2_ systems are unstable for concentrations *x* < 0.35, as shown in Fig. 5. Therefore, the large magnitudes of the potential of 1T-Na_*x*_MoS_2_ compounds for low concentrations are unlikely to use practically. Our results show that the average potential of 1T-Na_*x*_MoS_2_ is obtained approximately as 1.28 V. Compared with Li-intercalated MoS_2_, the average potential of Na_*x*_MoS_2_ is much lower because of the weaker binding of Na atoms. Since ideally a good anode should have a low electrode potential, our calculated voltage profile suggests that layered MoS_2_ is suitable as an anode for an NIB. When this Na-intercalated MoS_2_ anode is combined with high-capacity cathode materials such as Na_3_MnPO_4_CO_3_, the Na-ion battery cell can yield a desirable open circuit voltage in the range of 2.5~3.5 V.

## Conclusions

In conclusion, we investigated the adsorption energies, phase transition for the adsorption of Na onto MoS_2_ monolayer, and electrochemical properties of Na_*x*_MoS_2_ by using the first-principles DFT method. The traditional trigonal prismatic 1H-MoS_2_ phase is stable under normal conditions. However, a comprehensive study of the relative phase stability of MoS_2_ tells us that the other structural phase transition can be stable by adsorption. Our results show that some triangular Mo-Mo clustering appears in 1H-Na_*x*_MoS_2_ with the increasing of Na-adsorption concentration. On the other hand, some diamond-like Mo-Mo chains appear in 1H-Na_*x*_MoS_2_ when the Na-adsorption concentration is beyond 25 %. What is more, the adsorption of Na on MoS_2_ induces a phase transformation at *x* = 0.35 from the 1H to 1T phase. Our calculated results show that the adsorption of Na onto MoS_2_ monolayer results in a lower energy barrier from 1H to 1T-MoS_2_. Finally, Na_*x*_MoS_2_ compound is likely to become a battery anode material with a low average electrode potential of 0.72~1.28 V.
